# Preferences and willingness-to-pay for a blood pressure telemonitoring program using a discrete choice experiment

**DOI:** 10.1038/s41746-023-00919-3

**Published:** 2023-09-25

**Authors:** Ian Yi Han Ang, Yi Wang, Shilpa Tyagi, Gerald Choon Huat Koh, Alex R. Cook

**Affiliations:** 1 Saw Swee Hock School of Public Health, National University of Singapore and National University Health System, Singapore, Singapore; 2 MOH Office for Healthcare Transformation, Ministry of Health, Singapore, Singapore

**Keywords:** Health care economics, Health services

## Abstract

This study aimed to elicit the preferences and willingness-to-pay for blood pressure (BP) telemonitoring programs. This study also investigated the different factors or participant characteristics that could influence preferences and choice behaviors. Participants with hypertension were identified from an online survey panel demographically representative of Singapore’s general population. Participants completed a discrete choice experiment (DCE) with 12 choice sets, selecting their preferred BP monitoring program differing on five attributes: mode of consultation, BP machine type (with Bluetooth or not), BP machine price, monthly fee, and program duration. The base reference population (male, married, higher income, more formal education years, full-time worker, aged 55 to <65 years, and digital skills score of 36) preferred teleconsultation over in-person consultation, Bluetooth feature, lower machine price, lower monthly fee, and shorter program duration. A subgroup of participants can be considered teleconsultation-resistant, and three demographic factors were associated with lower preference for teleconsultation: female, fewer formal education years, and lower income. Considering the reference population and Bluetooth attribute, participants were willing to pay 66 SGD (~49 USD) additional for the machine to obtain the Bluetooth feature. Considering the reference population and teleconsultation attribute, participants were willing to pay 6.80 SGD (~5.10 USD) extra monthly fee for a program using teleconsultation. Here we report that amongst participants with hypertension, there is strong preference for the use of teleconsultation and a BP machine with Bluetooth feature in a BP monitoring program. However, a subgroup of participants are teleconsultation-resistant and would prefer in-person consultation.

## Introduction

The global disease burden continues to grow with ageing populations and rising prevalence of chronic diseases^1–4^. Health systems have immense pressures to cope with the increasing demand for health services^5^. Clinicians face increased workload and time stresses^6,7^, while patients face long wait times and low consultation time with their healthcare providers^8,9^. There is thus a need for health systems to optimize the use of healthcare manpower and resources and to improve patient care and experience.

Telehealth can help ease the increasing pressures of the health systems and be used to support chronic disease care management^10–12^. Patients require regular consultations with their chronic disease care provider to ensure the monitoring of disease progression and control, as well as for medication titration. In-person visits could be burdensome and inefficient, taking up patients’ time and causing potential income loss, with long wait times for a short consultation session^10,13,14^. The use of teleconsultation for regular reviews and check-ins could thus replace in-person visits without losing quality of care.

With the advent of COVID-19, the use of telehealth services has seen an increase in popularity and adoption^15–18^, as it reduces the risk of infection by leaving home and being near the care provider and other patients in the waiting area^19^. COVID-19 has also created a shift in knowledge, attitudes, and behaviors toward the use of telehealth services, and could thus provide a timely catalyst for new telehealth programs. In recent years, one such telehealth program that was piloted by the Ministry of Health Office for Healthcare Transformation (MOHT) in Singapore is the Primary Tech Enhanced Care (PTEC) home blood pressure (BP) monitoring program for those with hypertension^20^.

Singapore had a ratio of 2.4 doctors per 1000 persons in 2018^21^, which is a marked improvement from the ratio of 1.7 from 7 years earlier^22^. However, this ratio for a city-state remains low compared to other high-income economies with larger populations and rural areas^22,23^. At the same time, hypertension has a high prevalence in Singapore, with about 1 in 5 residents aged 30–69 years with BP ≥ 140/90 mmHg (18.9% in 2010, 21.5% in 2017)^24^. This prevalence is expected to rise by over 60% by 2040^25^. At public hospitals’ specialist outpatient clinics and polyclinics, which are large public primary care centers, waiting times have been on the rise due to the increasing patient load, particularly those seeking care in managing their chronic diseases. Time spent per patient is also low, with physicians in polyclinics spending less than 10 min of consultation time for 89% of chronic cases^26^.

Improved hypertension management is key in preventing disease worsening and complications, thereby decreasing the disease burden for the patient and the health system. From a health system and health policy perspective, the use of telehealth, be it with teleconsultations and/or telemonitoring, could significantly improve hypertension management. Understanding which attributes of such a telehealth program are preferred by patients with hypertension would thus be key in the design, implementation, and scaling up of such programs^12^.

Health preference research provides insights into trade-offs that help inform healthcare service providers on optimal design and pricing^27^. Using a discrete choice experiment (DCE) administered to participants with hypertension, this study aimed to elicit the preferences and willingness-to-pay for BP telemonitoring programs. In addition, patient populations are not homogenous, and different factors or characteristics of patient subgroups could influence the different preferences and choice behaviors. This study thereby aimed to investigate these subgroup differences.

In summary, this study demonstrates that overall, amongst participants with hypertension, there is a strong preference for the use of teleconsultation and a BP machine with Bluetooth feature in a BP monitoring program. Participants with hypertension are willing to pay about 5 SGD more monthly for teleconsultation and 66 SGD more for the Bluetooth feature as part of the BP telemonitoring program. However, there is a subgroup of participants (i.e., female, lower income, and fewer formal education years) who are teleconsultation-resistant, and would prefer in-person consultation instead. With the advent of COVID-19, there has been increased use of and exposure to telehealth services and programs, thereby strengthening the acceptance of and preferences for the use of teleconsultation.

## Results

Of the 229 participants in the online research platform that were invited to participate in this online survey, 216 started the survey, and 9 of them (all completers) indicated “No” to the question of being diagnosed with hypertension by a doctor. Of the 207 remaining eligible participants, 193 participants completed the full survey with the DCE section consisting of 12 choice sets. Summary statistics of the participants are presented in Table 1. The number of females and males was similar. Close to 38% of participants were aged 65 years or above, which was the retirement age in Singapore. About three-quarters of the participants were married. Close to 39% had fewer formal education years (11 years or less), and close to 38% of participants had full-time employment. Over one-quarter of participants were classified as with lower income (income below 3000 SGD, or those living in a 1-room public housing). The digital skills score, which was calculated with a summation (reverse scored) of the 10 adapted^28^ 5-point Likert scale questions on the mastery in the use of digital technologies (Supplementary Fig. 1), had a mean of 35.1 (higher the better).

**Table 1:** Baseline demographic of participants.

	Frequency	Percentage
Gender		
Male	100	51.8%
Female	93	48.2%
Ethnicity		
Chinese	170	88.1%
Malay, Indian, and others	23	11.9%
Age group		
Below 55 years	52	26.9%
55 years to below 65	68	35.2%
65 years and above	73	37.8%
Marital status		
Married	142	73.6%
Single, divorced, or widowed	51	26.4%
Education years		
Fewer formal education years	75	38.9%
More formal education years	118	61.1%
Employment		
Full-time	73	37.8%
Part-time or not working	120	62.2%
Income group		
Lower income	49	25.4%
Higher income	144	74.6%
	Mean (min, median, max)	SD
Digital skills score	35.1 (10, 36, 50)	9.1

The baseline demographic of the participants in the study are presented as a frequency and percentage of the total number of participants. The mean and standard deviation (SD) of the digital skills score of all the participants are also presented, along with the minimum (min), median, and maximum (max) scores.

A tabulation of the attributes and levels of the DCE is presented in Table 2. These attributes of the DCE were all features of a program within MOHT’s control to optimize: the mode of consultation, BP machine type (with Bluetooth or not), BP machine price, monthly fee, and duration of the program. All the levels of these attributes had positive chances of being chosen when they were presented to the participants as hypothetical choices (Table 2), suggesting that participants had to consider the trade-offs across different attributes. In about 50% of the choice tasks, participants chose not to enroll in the selected program. For the price of the machine, the monthly fee, and the duration of the program, the percentage of selection decreased as the values of these attributes increased to become longer or more expensive. Furthermore, the pattern of the percentages suggested that these attributes should be entered into the model as continuous variables. For example, as the duration of the program increased from 1 to 2 years, and to 3 years, the percentage of selection dropped from 55 to 50%, and then to 45%.

**Table 2:** Appearance and selection frequencies of the discrete choice experiment.

Attributes and levels	Total no. of appearances	Total no. of selection	Percentage of selection
Consultation mode			
Teleconsult	2315	1096	47.3%
In-person	2317	1220	52.7%
BP machine type			
Bluetooth BP machine	2312	1223	52.9%
Non-Bluetooth BP machine	2320	1093	47.1%
BP machine price			
$50	1544	930	60.2%
$100	1541	767	49.8%
$150	1547	619	40.0%
Monthly fee			
$5	1551	1017	65.6%
$10	1540	791	51.4%
$20	1541	508	33.0%
Minimum duration			
1 year	1534	846	55.1%
2 years	1552	782	50.3%
3 years	1546	688	44.5%
None	2316	1149	49.6%

The appearances and selection frequencies (as total number and percentage of the appearances) for the different levels of the attributes used in the discrete choice experiment are presented.

Table 3 shows the preference weights from the mixed logit model analysis. There was no evidence of left-and-right bias in choice selection. The reference population preferred teleconsultation over in-person consultation (coefficient = 1.12, 95% CI = 0.79 to 1.44, *P* < 0.001) and Bluetooth feature (coefficient = 0.92, 95% CI = 0.64 to 1.20, *P* < 0.001). Lower machine prices (coefficient = –0.01, 95% CI = –0.02 to –0.01, *P* < 0.001), lower monthly fee (coefficient = –0.16, 95% CI = –0.18 to –0.15, *P* < 0.001), and shorter duration of program (coefficient = –0.34, 95% CI = –0.44 to –0.25, *P* < 0.001) were preferred. The magnitude of the coefficient of the monthly fee was about 12 times that of the machine price, which suggests that the participants converted the monthly fee into yearly cost when they made their decisions. Random preference heterogeneity at the individual level was significant for all the levels.

**Table 3:** Mixed logit model results.

	Coefficient	95% CI	*P* value
Left	–0.12	(–0.25 to 0.01)	0.080
Base reference population			
None option	–0.37	(–0. 85 to 0.11)	0.134
Teleconsultation	1.12	(0.79 to 1.44)	<0.001
Bluetooth	0.92	(0.64 to 1.20)	<0.001
Machine price	–0.01	(–0.02 to –0.01)	<0.001
Monthly fee	–0.16	(–0.18 to –0.15)	<0.001
Duration	–0.34	(–0.44 to –0.25)	<0.001
Impact of demographic factors			
None option * female	–0.22	(–0.65 to 0.21)	0.319
None option * age above 65	0.45	(0.02 to 0.89)	0.042
None option * not married	0.96	(0.45 to 1.47)	<0.001
None option * fewer education years	–0.70	(–1.10 to –0.30)	<0.001
None option * digital skills score	0.06	(0.03 to 0.08)	<0.001
Teleconsultation * female	–0.62	(–0.95 to –0.30)	<0.001
Teleconsultation * age below 55	0.10	(–0.23 to 0.42)	0.560
Teleconsultation * not married	–0.26	(–0.61 to 0.10)	0.158
Teleconsultation * fewer education years	–0.89	(–1.19 to –0.59)	<0.001
Teleconsultation * not full-time employment	–0.07	(–0.38 to 0.23)	0.649
Teleconsultation * lower income	–0.53	(–0.88 to –0.19)	0.003
Bluetooth * age below 55	–0.44	(–0.78 to –0.10)	0.011
Bluetooth * age above 65	–0.27	(–0.61 to, 0.07)	0.121
Bluetooth * not full-time employment	–0.11	(–0.41 to 0.19)	0.456
Machine price * age below 55	–0.005	(–0.009 to –0.001)	0.008
Monthly fee * lower income	0.05	(0.02 to 0.08)	<0.001
Monthly fee * digital skills score	–0.002	(–0.003 to 0)	0.036
Random preference heterogeneity at the individual level			
None option	4.78	(4.27 to 5.28)	<0.001
Teleconsultation	2.48	(2.22 to 2.74)	<0.001
Bluetooth	0.86	(0.69 to 1.03)	<0.001
Machine price	0.005	(0.004 to 0.007)	<0.001
Monthly fee	0.10	(0.09 to 0.12)	<0.001
Duration	0.58	(0.49 to 0.68)	<0.001

Mixed logit model results with control of demographic factors are presented, along with the 95% confidence intervals (95% CI) of the coefficient. The *P* values presented are for the *t* test for the significance of the coefficients in the mixed logit model. The base reference population were male; married; higher income; more formal education years; full-time worker; age at 55 years to below 65; and with a digital skills score of 36 (median). The interaction of demographic factors with the levels of the DCE attributes are denoted with an asterisk (*).

The coefficient for Bluetooth of participants aged below 55 years is –0.44 (95% CI = –0.79 to –0.10, *P* = 0.011), i.e., 0.44 lower compared to that of the reference population participants, which is 0.92, thereby equating to a preference weight of 0.48 (0.92 subtracting 0.44). Participants aged below 55 years had preference weight coefficients lower than that of the reference population participants for machine price. Participants with lower income and participants with lower digital skills score had preference weight coefficients lower in absolute terms than that of the reference population participants for monthly fee.

Comparing teleconsultation with in-person consultation, three factors were associated with lower preference for teleconsultation: being female, having fewer formal education years, and having a lower income. In our overall study sample, there is a significantly greater portion of females with fewer formal education years than males (*P* = 0.020). This difference was found between females and males that are 65 years old and above (*P* = 0.02), but not between females and males that are below 65 years old (*P* = 0.300). The preference weight coefficients for teleconsultation of female participants, participants with fewer formal education years, and lower-income participants, are 0.63, 0.89, and 0.53 lower, respectively, than that of the reference population participants, which is 1.12. The preference weights for teleconsultation of female participants, participants with fewer formal education years, and lower-income participants, thus equate to 0.49, 0.23, and 0.59, respectively. For participants with these three characteristics, their preference weight was –0.32 for teleconsultation. Hence, combining these three characteristics, this formed a subgroup of participants that were female, with fewer formal education years, and lower income, named “Low SES Female”, who preferred face-to-face consultation over teleconsultation.

**Fig. 1:**
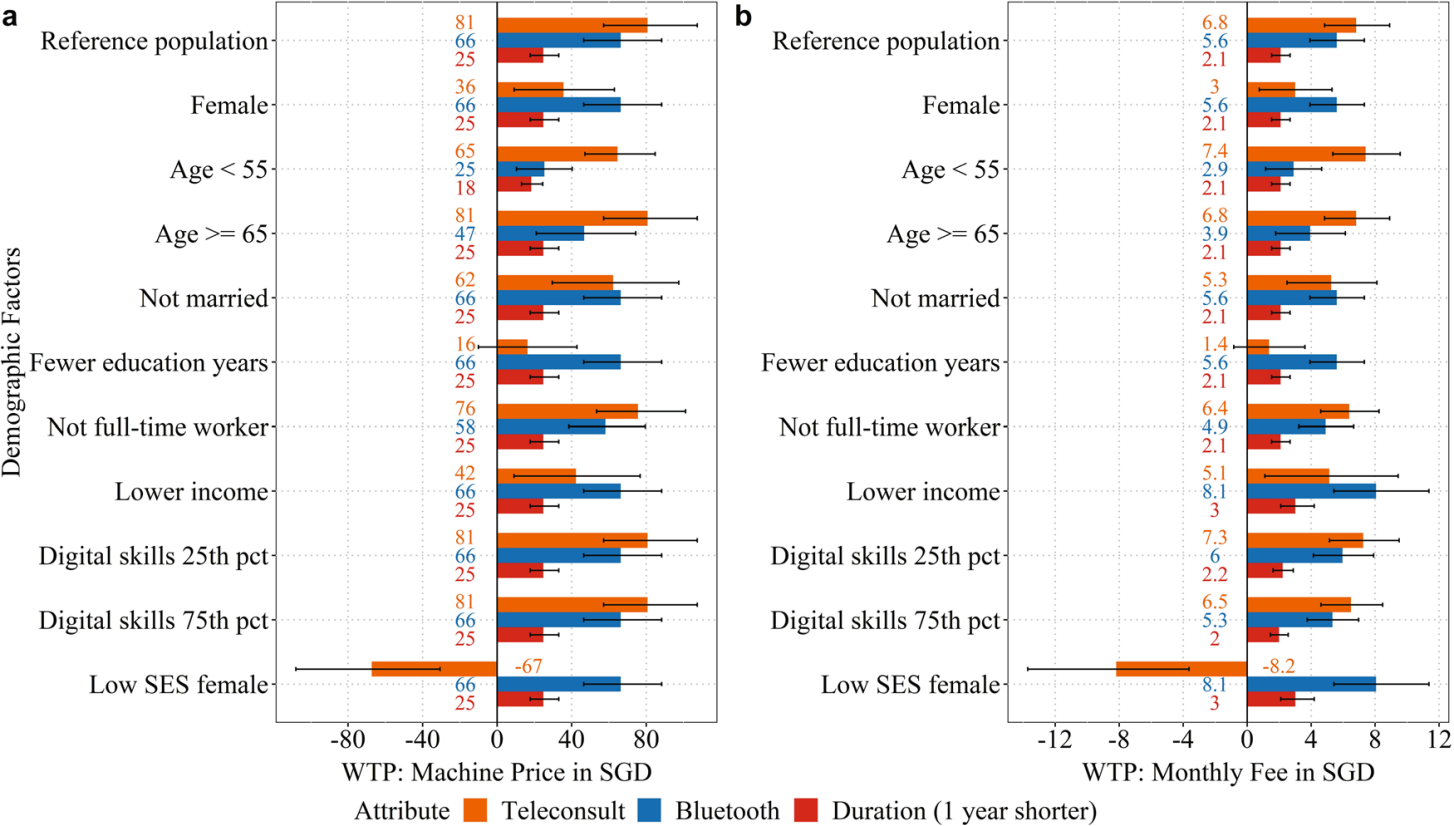
Willingness-to-pay. Willingness-to-pay for the program using teleconsultation, Bluetooth feature of BP machine, or 1 year shorter duration of the program measured by **a** the price of blood pressure (BP) machine and **b** the monthly fee. The base “reference population” comprise of those who were male, married, with higher income, with more formal education years, full-time worker, aged at 55 years to below 65 years, and with a digital skills score of 36 (median). Digital skills score was calculated with a summation (reverse scored) of the 10 adapted 5-point Likert scale questions on the mastery in the use of digital technologies, and a higher score is indicative of having higher mastery. Digital skills score at the 25th percentile (“digital skills 25th pct”) and at the 75th percentile (“digital skills 75th pct”) have a 6- and 5-point difference from the median score. The “low SES female” comprise of subgroup of participants that were female, with fewer formal education years, and lower income. Error bars indicate the 95% confidence intervals of the willingness-to-pay.

Willingness-to-pay, in 2020 values, with 95% confidence interval are presented in Fig. 1. Panel A uses the price of BP machine to calculate the willingness-to-pay. Considering the reference population and Bluetooth attribute, the participants were willing to pay 66 SGD (~49 USD) additional for the BP machine to obtain the Bluetooth feature. Panel B uses the monthly fee to calculate willingness-to-pay. Considering the reference population and teleconsultation attribute, the participants were willing to pay 6.80 SGD (~5.10 USD) additional for monthly fee for a program using teleconsultation. The bars for the different demographic factors show the willingness-to-pay by changing that demographic factor while all other demographic factors are held fixed like the reference population. For example, the bars for female show the willingness-to-pay of female participants that are also aged at 55 years to below 65 years, married, with more formal education years, full-time worker, with higher income, and with digital skills score at the median of the sample (score of 36). This is except for the combination subgroup “Low SES Female”, which is a combination of multiple demographic factors.

Willingness-to-pay for Bluetooth and the duration of the program were similar across different groups. However, willingness-to-pay for teleconsultation was different across different groups; and notably, participants with lower income and fewer formal education years had a lower preference weight for teleconsultation. The “Low SES Female” group could be considered to be teleconsultation-resistant. This group was willing to pay 67 SGD (~50 USD) more for the machine, or 8.20 SGD (~6.10 USD) more for the monthly fee, if they could switch from teleconsultation to in-person consultation.

**Fig. 2:**
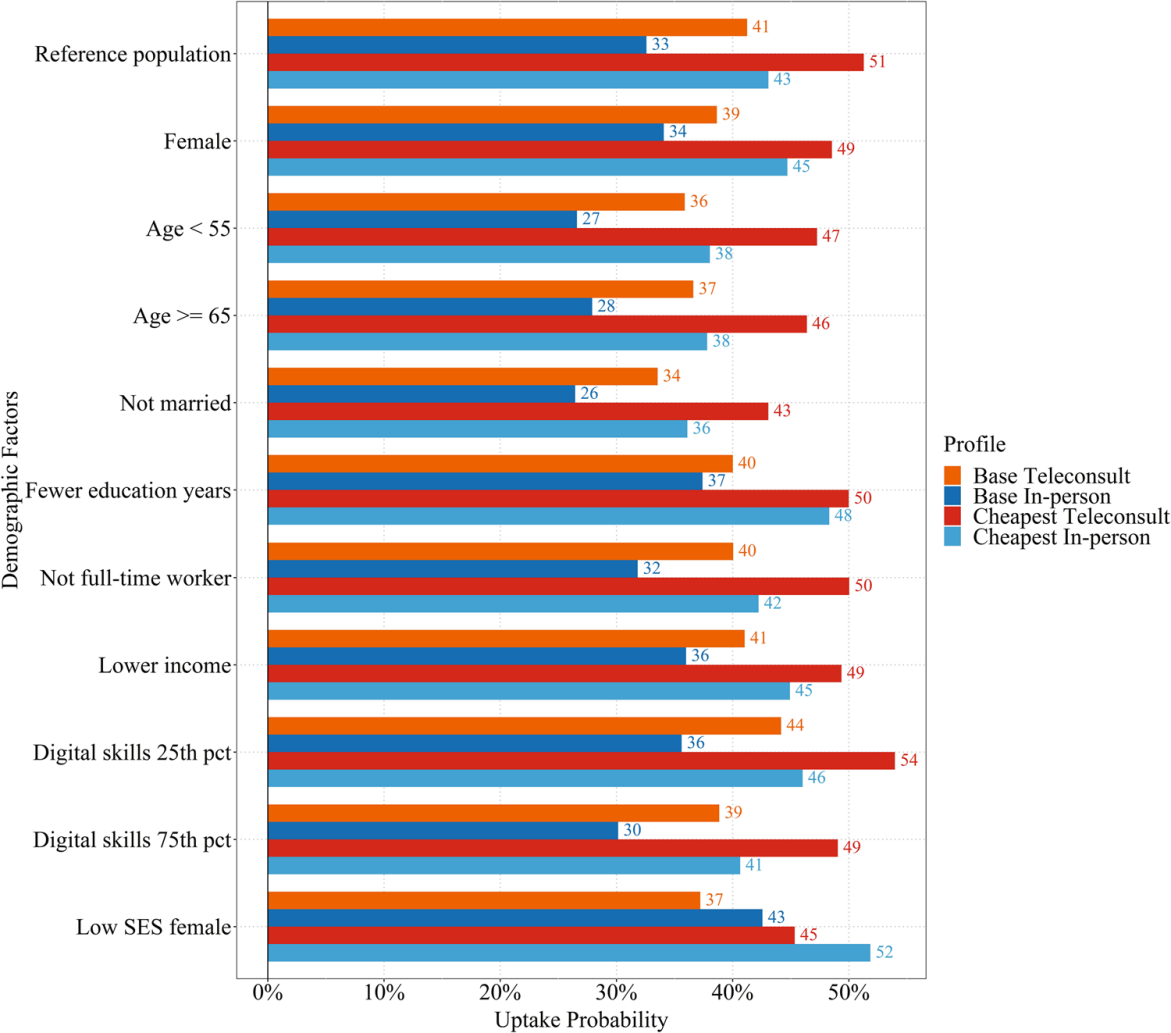
Program uptake probability. Uptake probability for the four different program profiles across the different demographic factors and participant subgroups. The base “reference population” comprised of those who were male, married, with higher income, with more formal education years, full-time worker, aged at 55 years to below 65 years, and with a digital skills score of 36 (median). Digital skills score was calculated with a summation (reverse scored) of the 10 adapted 5-point Likert scale questions on mastery in the use of digital technologies, and a higher score is indicative of having higher mastery. Digital skills score at the 25th percentile (“digital skills 25th pct”) and at the 75th percentile (“digital skills 75th pct”) have a 6- and 5-point difference from the median score. The “low SES female” comprised a subgroup of participants that were female, with fewer formal education years, and lower income.

Program uptake probabilities are presented in Fig. 2. We considered the following program profiles: (1) Base Teleconsult: teleconsultation, Bluetooth machine, machine price at 100 SGD, monthly fee at 10 SGD, mandatory program duration of 2 years; (2) Base In-Person: in-person consultation, Bluetooth machine, machine price at 100 SGD, monthly fee at 10 SGD, mandatory program duration of 2 years; (3) Cheapest Teleconsult: teleconsultation, Bluetooth machine, machine price at 50 SGD, monthly fee at 5 SGD, mandatory program duration of 1 year; (4) Cheapest In-Person: in-person consultation, Bluetooth machine, machine price at 50 SGD, monthly fee at 5 SGD, mandatory program duration of 1 year.

The bars for the different demographic factors show the uptake probability by changing one demographic factor at a time, except again for the combination subgroup “Low SES Female”. For the first two program profiles, the uptake probability ranged between 26 and 44%. In general, participants had higher chances of participating in the program if it was teleconsultation. For the Low SES Female group, however, the uptake probability is higher by around 6–7% for a program with in-person consultation. The third and fourth program profiles achieved the highest uptake probabilities, ranging between 36 and 56%. Overall, comparing the different program profiles, when the machine price decreases by 50 SGD, the monthly fee decreases by 5 SGD, and the program duration decreases by 1 year, the uptake probability consistently increased by 8–11%.

## Discussion

The main finding that the base reference population preferred teleconsultation to in-person consultation, and valued having a Bluetooth feature in the BP machine, supports a large-scale adoption of a BP telemonitoring program for patients with hypertension. This finding corroborates recent findings on the positive receptivity for the use of teleconsultation and telemonitoring after the advent of COVID-19^15–18^, which has led to more prominent widespread use of teleconsultation and a strong demonstration of its utility. Previous studies conducted before COVID-19 have also found positive attitudes for the use of BP telemonitoring in stroke patients^29,30^. Unsurprisingly, this study found that a lower BP machine price, lower monthly fee, and shorter duration of commitment to the program was preferred over more expensive and longer programs.

In our study, three demographic factors were associated with a lower preference for teleconsultation: being female, having a lower income, and having fewer years of formal education. In our overall study sample, there was a significantly greater portion of females with fewer formal education years than males. This difference was driven by those that are 65 years old and above, which is expected as education opportunities were lower for females in the older generations. The demographic factor of older age group of 65 years and above alone was not found to influence the willingness-to-pay. As such, the teleconsultation-resistant group remains this intersection of the three demographic factors of being female, having a lower income, and having fewer years of formal education.

Some studies have found that the factor of female alone have higher preference^31^ or did have higher actualization^14,32–34^ of teleconsultation appointments, likely because they found teleconsultation convenient and time-saving. However, earlier studies have also shown that the combination of female, lower income, and with fewer formal education years were associated with lower access to the use of technology^35,36^. In our study, we found that the subgroup with this combination of these factors was willing to pay 67 SGD (~50 USD) more for the machine, or 8.20 SGD (~6.10 USD) more for the monthly fee if they could switch from teleconsultation to in-person consultation. Perhaps this particular subgroup has lesser access and exposure to teleconsultation, and thus less likely to discover the potential benefits that teleconsultation could bring. Nonetheless, the uptake probability for the cheapest Teleconsult program is similar to that of the base In-person program in this subgroup, indicating cost playing a more crucial role than just the mode alone.

A major implication from the findings is that equitable considerations have to be made in the care and management of patients with hypertension. The population of those with hypertension is not homogenous, with clear preferential differences differing for those of different demographic profiles. Even though this study elucidates an optimized design of a BP telemonitoring program that will maximize program uptake given the budget constrain from the government, the reality is that variations will have to exist to cater to those who would prefer a BP monitoring program with in-person consultation. Furthermore, the random preference heterogeneities at the individual level were significant for all the levels. These individual level heterogeneities could capture other factors affecting people’s preference but were not controlled in this study. Identifying all these factors can be costly and not feasible. In practice, the government could design a program which allows personal customization by patients themselves.

The program design being tested in our study has two financial components: machine price and monthly fee. While machine price is straightforward to consider, the monthly fee needs another step of calculations to understand the total cost. Our results showed that participants with lower digital skills score and participants with lower income were less sensitive (had lower disutility) to an increase in monthly fee. Participants with higher digital skills score may have experience with many free digital services online, and hence were more sensitive to the monthly fee. For participants with lower income, they had lower sensitivity to monthly fees but not machine price. This could be influenced by how clearly the participants understood the DCE task with the preamble (Supplementary Methods). Participants with lower income might have lower financial literacy and require additional steps to understand the full financial implication of such monthly fees. The correlation of low financial literacy with ineffective spending and financial planning has been well documented^37^. Numeracy in some population groups in the United States and other countries is low and these people have been found to make mistakes in financial decisions requiring simple calculations^38^. Hence, assisting patients in the understanding of the full financial implication of such health programs with complex cost components is important.

One of the key strengths of this study was the use of a DCE, which elicits preferences indirectly through choice selection, as opposed to direct reporting or ranking. Participants might not always declaratively know which factors they prefer or be able to quantify how important each factor is to them. Through the use of a DCE, how strongly the various attributes are as preferences can be elicited, and a quantifiable utility value generated. Another strength of this study was the timely investigation into preferences for telehealth programs as they become more mainstream after increased use and exposure due to COVID-19.

One of the limitations of this study was that the participants from the SPHS Online Panel were all able to read English, had access to the internet via a computer or smartphone and could use it to complete the survey. This meant that the participant pool would not have represented those unable to read English, and had no access or ability to use a computer or smartphone. However, it should be noted that the Singapore population has a high English literacy rate^39^ and a high percentage with access to the internet^40^, and so this limitation does not severely limit the generalizability of the participant cohort, although the findings may not generalize to other settings where internet access may act as a barrier to uptake of telemedicine. We also did a comparison of the demographic profile of our study sample (Supplementary Table 1) with that of a separate published study in Singapore that investigated the demographic profiles of those with hypertension using a multi-ethnic cohort in Singapore^41^. Compared to this cohort, our study sample has a smaller proportion with fewer years of formal education, smaller proportion with lower income, and are older. However, we cannot identify which sample is more representative of the patients with hypertension in Singapore, since the multi-ethnic cohort has a much lower proportion of Chinese than that in the general Singapore population.

Another limitation was that the participant pool was selected based on self-reporting of hypertension, with no additional verification or use of medical records. However, participants reported this diagnosis of hypertension as part of reporting on the chronic diseases that they have, without knowing that there would be a follow-up survey with hypertension as a criterion for selection. Therefore, there was a very low likelihood of false reporting in order to participate in this study. Those without an official clinical diagnosis of hypertension or had wrongly reported so before proceeded with this study survey and were identified with verification questions to filter their data out from analyses.

## Methods

### Study design and participants

The study was a cross-sectional online survey administered through the Singapore Population Health Studies (SPHS) Online Panel, approved by the National University of Singapore Institutions Review Board (NUS-IRB; Ref No.: H-18-011). This panel served as an online research platform that facilitated public health research on a diverse range of topics, through monthly surveys related to public health. When the survey was conducted, the panel was made up of 2413 active participants who were demographically representative of the general population of Singapore. Participants had already been recruited into the SPHS Online Panel and provided informed consent, hence no further recruitment was conducted. This study obtained a waiver for documented informed consent from the NUS-IRB (Ref No.: NUS-IRB-2020-82).

The survey was designed as a follow-up for participants who had indicated having hypertension in a separate survey by the research team administered four months prior (229 out of 1975). Of the 229 participants invited to participate in this survey, 216 started it, and 202 completed it. Nine participants (all completers) indicated “No” to the question on being diagnosed with hypertension by a doctor, and so their data were removed.

### Procedure**s**

Participants in the panel who met the above criteria for this survey received a notification by email and/or SMS about the survey in December 2020. Participants accessed the survey via the provided link and completed the survey on the Research Electronic Data Capture platform. The survey had an expiration date for completion within 10 days and was no longer accessible once it had expired. Before proceeding, participants were provided a participant information sheet. The survey had two parts: an initial 45 questions covering demographics, healthcare use, digital use, digital skills, and their hypertension management, followed by the DCE with 12 choice sets.

### Selection of attributes for DCE

The use of DCEs is common in health preference research^42,43^ and has been found to have moderate predictive value of real-world choice behaviors^44^. MOHT was developing and implementing the PTEC home BP monitoring program for those with hypertension^20^, and was a key stakeholder for this study. MOHT was interested in investigating the optimal combinations of controllable attributes to roll out a sustainable scaled-up program.

The program by design would use teleconsultation in combination with a BP machine that has Bluetooth capabilities to automatically synchronize the readings for remote monitoring. For the program to be cost-effective and for the impact of the program to take hold, the design of the program would require patients’ commitment to enroll in the program for a fixed duration. Thus, the price of the BP machine and the monthly fee of the program were factors that MOHT was interested in investigating. The attributes chosen for the DCE were all features of such a program within MOHT’s control to optimize: the mode of consultation, BP machine type (with Bluetooth or not), BP machine price, monthly fee, and duration of the program.

### DCE design

**Fig. 3:**
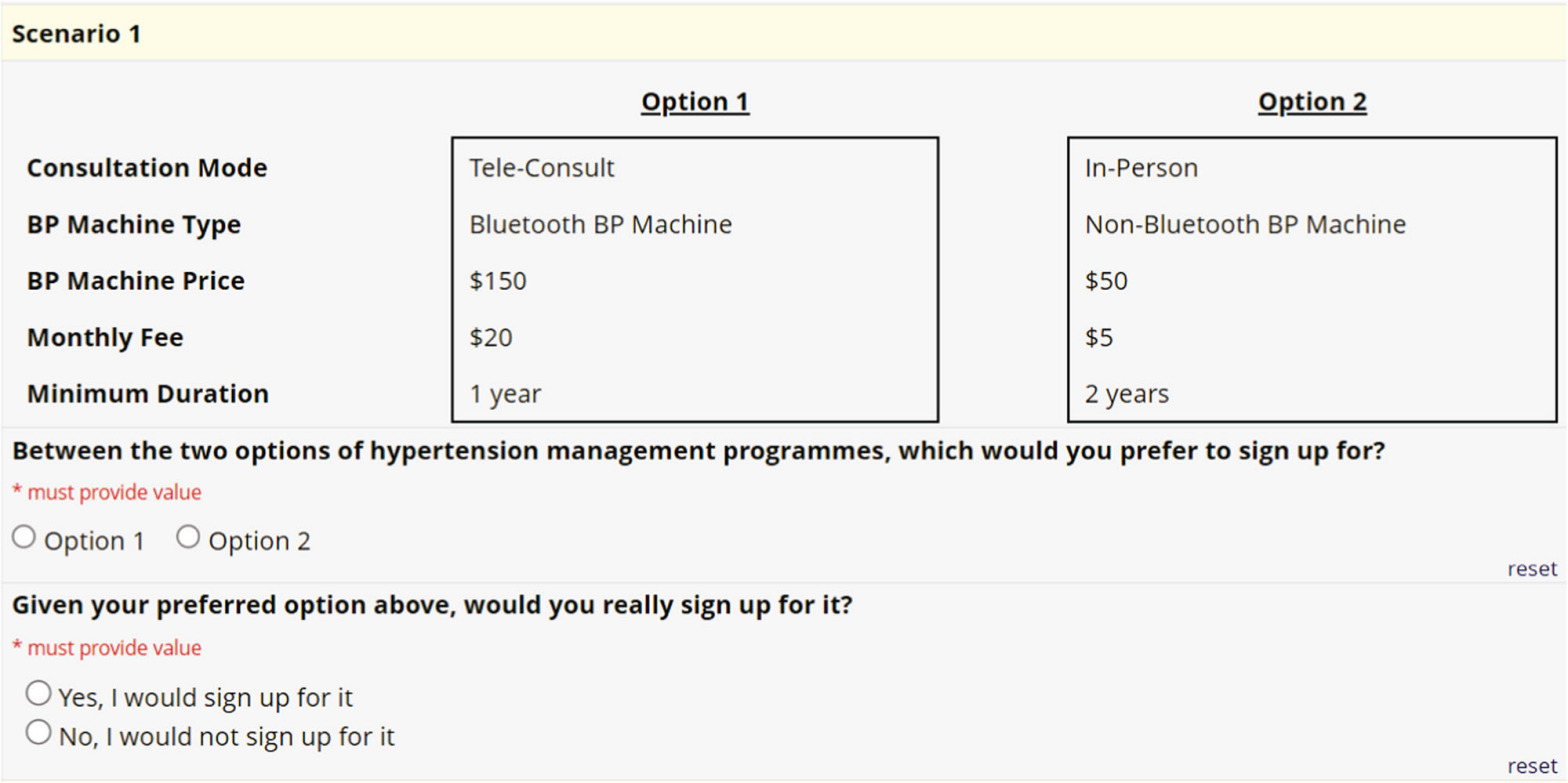
DCE choice task. An example of one of the choice task questions in the discrete choice experiment.

The final set of five attributes and levels is presented in Table 2. The DCE questionnaire was designed using Sawtooth Software Inc.’s Lighthouse Studio version 9.8. A two-stage design was used. The questionnaire was designed using the Balanced Overlap option provided by Lighthouse Studio, with only the main effects considered. Twenty blocks were used, with twelve tasks per block. A simulation exercise was conducted to examine the coverage matrix of the design and test the sample size required. An example of a DCE choice task is presented in Fig. 3.

### Statistical analysis

Mixed logit model was used in the analysis to incorporate two types of preference heterogeneities: (1) preference heterogeneity that the demographic factors can explain and (2) random preference heterogeneity at the individual level. Equation (1) is the formula for the random utility ${U}_{{ij}}$ of individual $i$, for a program $j$, where ${x}_{{ij}}$ is a vector describing the details of the consultation program and ${\epsilon }_{{ij}}$ is the idiosyncratic error term assumed to follow a type 1 extreme value distribution. Equation (2) is the formula for the ${\beta }_{i}$ term in Eq. (1). The ${{Demo}}_{i}$ term is a vector including the demographic factors in the analysis. The ${\beta }_{{Demo}}$ term shows the preference heterogeneity that is affected by demographic factors. The ${\eta }_{i}$ term follows multivariate normal distribution representing the random preference heterogeneity which includes all unobserved factors that affect individuals’ preferences.

1 $${U}_{{ij}}={\beta }_{i}\times {x}_{{ij}}+{\epsilon }_{{ij}}$$

2 $${\beta }_{i}=\beta +{{\beta }_{{Demo}}\times {Demo}}_{i}+{\eta }_{i}$$

Consultation mode and BP machine type were entered into the model as discrete variables. BP machine price, monthly fee, and minimum duration were entered into the model as continuous variables based on the tabulation pattern of the levels of each attribute and the percentage of selection. Bayesian information criterion (BIC) was compared between the model using continuous values and the model using discrete values for the three attributes of BP machine price, monthly fee, and minimum duration, one variable at a time. Smaller BICs were obtained for all models using continuous values compared to models using discrete values, confirming that these three attributes should be entered into our models as continuous variables.

Demographic factors that were entered into the model included gender, age group, marital status, education years, employment, income group, and digital skills score measuring the mastery that participants have with use of digital technologies such as smartphones. Age was classified into three categories: 65 years and above (the retirement age in Singapore), 55 years to below 65 years, and below 55 years. The choice of 55 years was chosen to ensure enough participants were in the lower age group for estimation. Marital status was classified into two categories: married and not married (single, divorced, or widowed).

Participants with education levels at secondary school or below were considered as having fewer formal education years (11 years or less), while participants with education levels above secondary school were considered as with more formal education years (more than 11 years). Employment status was classified into two categories: full-time and not full-time (part-time or not working). Information on household income and housing type were considered together to generate the two categories of income level: lower income and higher income. Participants with monthly household income below 3000 SGD, or those living in a 1-room public housing were categorized as lower income. The digital skills score was derived from a set of ten questions adapted from ref. ^28^, with participants rating on a 5-point Likert scale their mastery with regards to various uses of digital technology (Supplementary Fig. 1). The score was calculated with a summation of the ten questions (reversed scored) and a higher score is indicative of having higher mastery. The digital skills score was modeled as continuous, but with the score rescaled with the subtraction of the median to allow for ease of interpretation.

To examine how demographic factors affected preferences, the demographic factors were entered into the model by interacting with the levels of the DCE attributes. There are 48 possible interaction terms with full interaction, which could lead to multi-collinearity issues given the sample size. Therefore, in the selection of the final model, to achieve a parsimonious specification, one demographic factor at a time was considered, as well as its full interaction with the levels. Only interaction terms with *P* lower than 0.10 were kept in the final model.

The preference weights coefficients of the interaction terms indicate the preference weight differences between the reference population and the population with that demographic factor switched (i.e., all other factors remaining the same as the reference population except for that demographic factor). The base reference population comprised of those who were male, married, with higher income, with more formal education years, full-time worker, aged at 55 years to below 65 years, and with a digital skills score of 36 (median). Latent Class Analyses were also conducted but not included in the main results (Supplementary Note 1 and Supplementary Table 2).

Willingness-to-pay was calculated and presented in SGD, and can be taken to be December 2020 values. The USD values presented were calculated using the historical exchange rate on 1 December 2020^45^. Krinsky and Robb’s method was used to generate the confidence intervals^46^. Uptake probability was calculated by considering a few desired program profiles. All statistical analyses were carried out using R^47^.

### Supplementary information

Supplementary Information

## Data Availability

The data collected and analyzed in this study are only available from the corresponding author upon reasonable request and with permission of the institution review board.
