# Supplementary material for: Preferences and willingness-to-pay for a blood pressure telemonitoring program using a discrete choice experiment
Source: NPJ Digit Med. 2023 Sep 25;6:176. doi: 10.1038/s41746-023-00919-3 (PMC10520087; doi:10.1038/s41746-023-00919-3)
Supplement: Supplementary file 1 — Supplementary Information [file 41746_2023_919_MOESM1_ESM.pdf]

Supplementary Information

Supplementary Figure 1. Digital Skills Questions

Questions for the digital skills score.

How well do you think you master the following activities:

1: Excellent
2: Good
3: Fair
4: Poor
5: Very Poor

	1	2	3	4	5	
**Sending / receiving emails**<br>* must provide value						reset
**Buying goods or services over the Internet**<br>* must provide value						reset
**Reading or downloading online news, newspaper or magazines**<br>* must provide value						reset
**Internet banking**<br>* must provide value						reset
**Accessing institutions / organizations (e.g. for getting information, filling e-forms, etc.)**<br>* must provide value						reset
**Playing or downloading games, images, films or music**<br>* must provide value						reset
**Listening to web radio or watching web television**<br>* must provide value						reset
**Telephoning or making video calls over the internet**<br>* must provide value						reset
**Social networking (e.g. Facebook, Twitter, etc.)**<br>* must provide value						reset
**Posting messages to chat sites, blogs or forums, or instant messaging (e.g. WhatsApp, WeChat, etc.)**<br>* must provide value						reset

Supplementary Methods.

Preamble for the discrete choice experiment survey section.

For this second part of the survey, there will be 12 questions.
For each question, you will be presented with 2 options and asked to choose the option that you prefer for the following scenario:

You have hypertension and you have decided to take action to monitor your blood pressure regularly and to try to keep it low. Your doctor has recommended for you to sign up for a hypertension management programme. Imagine that you do not have a working home blood pressure (BP) machine and so need to buy a new one. There are two different types of BP machines, Non-Bluetooth or Bluetooth, and there are two different consultation modes, In-Person or Tele-consult. This creates four different combinations of how the hypertension management programme is run.

- **In-Person with Non-Bluetooth BP Machine:**
  This in-person programme consists of going to the clinic to meet with the care team once a month. Using your non-Bluetooth BP machine, you will take your blood pressure at least once a week, manually record the readings and bring them to your visit with the care team. The care team will review the blood pressure readings recorded and provide advice and personalised education on lifestyle changes when you meet in-person.

- **In-Person with Bluetooth BP Machine:**
  This in-person programme consists of going to the clinic to meet with the care team once a month. Using your Bluetooth-enabled BP machine, you will take your blood pressure at least once a week, and the machine will automatically record the readings in the vital signs monitoring app on your phone. The care team will review the blood pressure readings recorded and provide advice and personalised education on lifestyle changes when you meet in-person.

- **Tele-consult with Non-Bluetooth BP Machine:**
  This tele-consult programme consists of a video call with the care team once a month, without having to go to the clinic. Using your non-Bluetooth BP machine, you will take your blood pressure at least once a week, manually input the readings in the vital signs monitoring app on your phone and send them to the care team. The care team will review the blood pressure readings recorded and provide advice and personalised education on lifestyle changes over video call. In addition, there is a chatbot (in English or Chinese), which provides timely and interactive advice through automated SMS. It can prompt you if you missed taking your blood pressure and can guide you towards appropriate self-care and encouragement towards a healthier lifestyle.

- **Tele-consult with Bluetooth BP Machine:**
  This tele-consult programme consists of a video phone call with the care team once a month, without having to go to the clinic. Using your Bluetooth-enabled BP machine, you will take your blood pressure at least once a week, and the machine will automatically record the readings in the vital signs monitoring app on your phone and send them to the care team. The care team will review the blood pressure readings recorded and provide advice and personalised education on lifestyle changes over

video call. In addition, there is a chatbot (in English or Chinese), which provides timely and interactive advice through automated SMS. It can prompt you if you missed taking your blood pressure and can guide you towards appropriate self-care and encouragement towards a healthier lifestyle.

These different programmes will all have different pricing for the BP machine, different monthly fee that you have to pay for the programme, and different minimum duration that you have to commit to the programme.

In the following questions, the exact same type of programme using the same approach and BP machine might charge differently for the BP machine and the monthly fee, and have a different minimum duration. Please assume that the quality of care remains the same for that type of programme in these different imaginary options.

An example of such a question is presented below:

Between the two options of hypertension management programmes, which would you prefer to sign up for?

	**Option 1**	**Option 2**
Consultation Mode	In-Person	Tele-consult
BP Machine Type	Non-Bluetooth BP Machine	Bluetooth BP Machine
BP Machine Price	$100	$150
Monthly Fee	$10	$5
Minimum Duration	3 years	1 year

Supplementary Table 1.

The demographic profiles of the participants in this study and those in the Multi-Ethnic Cohort by Liew et al (2019).

		This Study	Multi-Ethnic Cohort
	**Total Number**	**193**	**3178**
**Gender**			
	Male	51.8%	47.1%
	Female	48.2%	52.9%
**Ethnicity**			
	Chinese	88.1%	47.8%
	Malay, Indian, & Others	11.9%	52.2%
**Age Group**			
	<40	3.1%	9.2%
	40-49	14.0%	25.1%
	50-59	28.0%	34.8%
	>=60	54.9%	30.9%
**Marital Status**			
	Married	73.6%	80.4%
	Single, divorced, or widowed	26.4%	19.6%
**Education Years**			
	Primary or lower	5.7%	42.0%
	Secondary	30.1%	40.0%
	Tertiary or higher	64.2%	18.0%
**Income Group**			
	<2000	27.2%	34.8%
	2000 – 3999	20.0%	32.2%
	>=4000	52.8%	33.0%

Supplementary Note 1.

Additional Latent Class Analyses (LCA) were conducted and the coefficients and their 95% confidence intervals (95% CI) presented in Supplementary Table 2.

BIC for 2 classes, 3 classes, 4 classes, and 5 classes were 4755, 4629, 4551, and 4553 respectively. Hence, 4 classes were chosen. Class I were teleconsultation resistant who preferred to participate in the program (None option = –4.07, 95% CI [–5.55, –2.59]) and preferred face-to-face consultation (Teleconsultation = –3.87, 95% CI [–4.75, –2.98]). Class II were teleconsultation supporter who preferred to participate in the program (None option = –2.19, 95% CI [–2.76, –1.61]) and preferred tele-consultation (Teleconsultation = 0.94, 95% CI [0.69, 1.19]). Class III were program supporter who preferred to participate in the program (None option = –4.49, 95% CI [–5.25, –3.73]) and were indifferent between tele-consultation and face-to-face consultation (Teleconsultation = 0.21, 95% CI [–0.02, 0.43]). Class IV were program resistant who preferred to not participate in the program (None option = 1.53, 95% CI [1.02, 2.03]) and preferred face-to-face consultation (Teleconsultation = –0.24, 95% CI [–0.39, 0.09]). The class share for the four classes in the sample were 9.1%, 21.1%, 21.5%, and 48.3%. The LCA results were not selected as the main results as the results of demographic factors can be difficult to interpret with more than two classes.

Supplementary Table 2. The results for the latent class analysis with 4 classes, with the 95% confidence intervals (95% CI) of the coefficients presented. The *P* values presented for the preference weights are derived from the t-test for the significance of those levels compared to the omitted reference levels in the latent class profile. The *P* values presented for the demographic factors are derived from the t-test for the significance of the coefficients compared to those of Class I, the reference class.

	**Class I Teleconsultation resistant**			**Class II Teleconsultation supporter**			**Class III Program supporter**			**Class IV Program resistant**		
	**Coefficient**	**95% CI**	***P* value**	**Coefficient**	**95% CI**	***P* value**	**Coefficient**	**95% CI**	***P* value**	**Coefficient**	**95% CI**	***P* value**
Left	0.03	–0.61, 0.67	0.927	0.06	–0.17, 0.30	0.590	0.11	–0.08, 0.31	0.246	–0.03	–0.17, 0.10	0.640
**Preference weight**												
None option	–4.07	–5.55, –2.59	<0.001	–2.19	–2.76, –1.61	<0.001	–4.49	–5.25, –3.73	<0.001	1.53	1.02, 2.03	<0.001
Teleconsultation	–3.87	–4.75, –2.98	<0.001	0.94	0.69, 1.19	<0.001	0.21	–0.02, 0.43	0.073	–0.24	–0.39, –0.09	0.002
Bluetooth	–0.05	–0.54, 0.43	0.831	0.56	0.33, 0.80	<0.001	0.41	0.18, 0.63	<0.001	0.17	0.02, 0.33	0.023
Machine price	–0.01	–0.02, –0.01	<0.001	–0.01	–0.02, –0.01	<0.001	–0.006	–0.01, 0.00	<0.001	–0.01	–0.01, –0.01	<0.001
Monthly fee	–0.12	–0.17, –0.07	<0.001	–0.13	–0.15, –0.11	<0.001	–0.064	–0.08, –0.05	<0.001	–0.09	–0.10, –0.08	<0.001
Duration	–0.23	–0.54, 0.09	0.157	–0.41	–0.56, –0.26	<0.001	–0.12	–0.26, 0.03	0.123	–0.22	–0.31, –0.12	<0.001
**Demographic factors**												
Constant	-	-	-	2.40	2.02, 2.79	<0.001	1.55	1.17, 1.94	<0.001	2.35	1.97, 2.74	<0.001
Female	-	-	-	0.62	0.28, 0.96	<0.001	0.87	0.54, 1.19	<0.001	0.75	0.44, 1.06	<0.001
Age < 55	-	-	-	–1.24	–1.72, –0.76	<0.001	–0.06	–0.52, 0.40	0.797	0.19	–0.26, 0.63	0.412
Age >= 65	-	-	-	–1.58	–2.03, –1.13	<0.001	–0.45	–0.86, –0.03	0.034	–0.74	–1.14, –0.34	<0.001
Not married	-	-	-	–0.86	–1.23, –0.48	<0.001	–0.40	–0.75, –0.05	0.024	–0.10	–0.43, 0.23	0.552
Fewer education years	-	-	-	–1.79	–2.14, –1.44	<0.001	–0.39	–0.71, –0.07	0.017	–1.01	–1.32, –0.70	<0.001
Not full-time worker	-	-	-	0.64	0.11, 1.17	0.018	–0.36	–0.88, 0.15	0.167	0.19	–0.32, 0.69	0.467
Lower income	-	-	-	–1.30	–1.69, –0.90	<0.001	–0.43	–0.78, –0.08	0.016	–0.55	–0.88, –0.22	<0.001
Digital skills score	-	-	-	–0.007	–0.03, 0.01	0.445	0.05	0.04, 0.07	<0.001	0.04	0.02, 0.05	<0.001
**Class membership**												
Share	9.1%			21.1%			21.5%			48.3%		
